# Epigenetic activities of flavonoids in the prevention and treatment of cancer

**DOI:** 10.1186/s13148-015-0095-z

**Published:** 2015-07-10

**Authors:** Christian Busch, Markus Burkard, Christian Leischner, Ulrich M. Lauer, Jan Frank, Sascha Venturelli

**Affiliations:** Division of Dermatologic Oncology, Department of Dermatology and Allergology, Medical University Hospital, Tuebingen, Germany; Department of Internal Medicine I, Medical University Hospital, Otfried-Mueller-Str. 27, 72076 Tuebingen, Germany; Institute of Biological Chemistry and Nutrition, University of Hohenheim, Stuttgart, Germany

**Keywords:** Epigenetics, HDAC, DNMT, Flavonoids, Phytochemicals, Nutrition, Cancer

## Abstract

Aberrant epigenetic modifications are described in an increasing number of pathological conditions, including neurodegenerative diseases, cardiovascular diseases, diabetes mellitus type 2, obesity and cancer. The general reversibility of epigenetic changes makes them an attractive and promising target e.g. in the treatment of cancer. Thus, a growing number of epigenetically active compounds are currently tested in clinical trials for their therapeutic potential. Interestingly, many phytochemicals present in plant foods, particularly flavonoids, are suggested to be able to alter epigenetic cellular mechanisms. Flavonoids are natural phenol compounds that form a large group of secondary plant metabolites with interesting biological activities. They can be categorized into six major subclasses, which display diverse properties affecting the two best characterized epigenetic mechanisms: modulation of the DNA methylation status and histone acetylation. High dietary flavonoid intake has strongly been suggested to reduce the risk of numerous cancer entities in a large body of epidemiological studies. Established health-promoting effects of diets rich in fruit and vegetables are faced by efforts to use purified flavonoids as supplements or pharmaceuticals, whereupon data on the latter applications remain controversial. The purpose of this review is to give an overview of current research on flavonoids to further elucidate their potential in cancer prevention and therapy, thereby focusing on their distinct epigenetic activities.

## Review

Cancer is one of the main causes of death worldwide, and cancer mortality is expected to be more than double in the next 20–40 years [1, 2]. In general, tumour growth is associated with both epigenetic and genetic aberrations resulting in altered gene expression [3]. Furthermore, epigenetic deregulation already occurs during early phases of neoplastic development and was suggested to have a comparable influence on promoting malignant transformation and subsequent tumour growth as genetic mutations [4]. For instance, DNA hypermethylation of promoter regions can cause binding of methyl DNA binding proteins, essential for gene inactivation (mainly of tumour suppressor genes), and global DNA hypomethylation is associated with chromosomal instability [5–7]. Both can be measured in cancer cells, and chromosomal instability is recognized as one of the “hallmarks of cancer” [7, 8]. Additionally, an altered histone acetylation status can modulate activation or silencing of tumour suppressor genes [9]. Despite the observation that epigenetic changes are heritable in somatic cells and epimutations are rare in non-transformed cells or healthy tissues, it is of interest to note that epigenetic modifications are potentially reversible. Therefore, targeting epigenetic mechanisms is a promising approach for cancer prevention and/or therapy and also for other diseases [5, 10, 11]. According to current estimates, cancer is in, at least, 30–40 % of the cases preventable with appropriate or balanced food and nutrition, regular physical activity and avoidance of obesity [2]. To date, multiple biologically active food components are strongly suggested to have protective potential against cancer formation, even though these effects are not yet firmly established for the majority of these compounds [12]. Examples are methyl-group donors, selenium, fatty acids, and phytochemicals, such as flavonoids, retinoids, isothiocyanates, and allyl compounds [2].

### Epigenetics and cancer

**Fig. 1:**
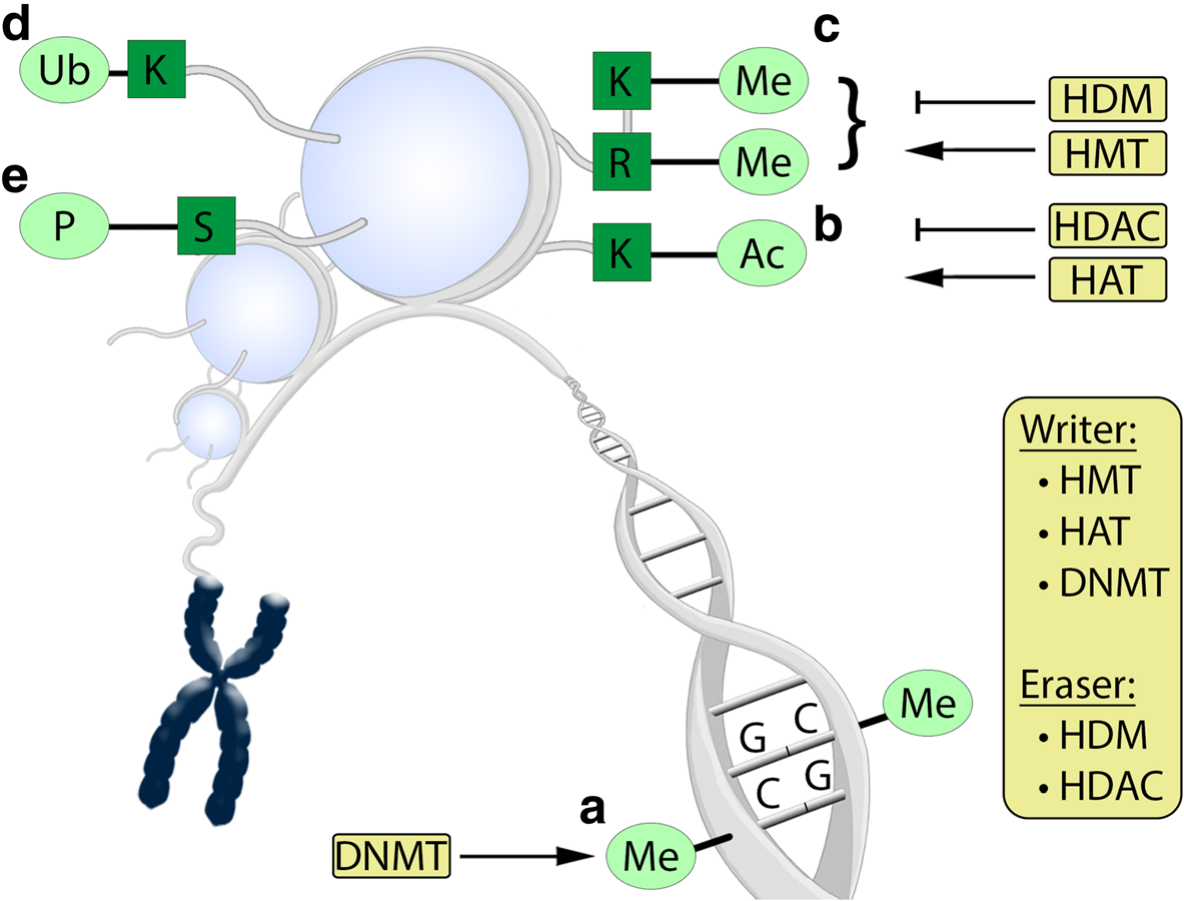
Important epigenetic modifications known to regulate gene expression. **a** DNA methylation of CpG islands in promoter regions by DNA methyltransferases (DNMT) represses gene activity. Posttranslational covalent histone modifications of lysine (K), arginine (R) or serine (S) residues in the “histone tail” also influence gene expression in different ways. **b** Histone acetylation (Ac) catalysed by histone acetyltransferases (HAT) is usually correlated to increased gene activity, whereas histone deacetylation caused by histone deacetylases (HDAC) is considered to decrease gene expression, even though histone hyperacetylation not always matches regions of increased gene activity. **c** Histone methylation (Me) and demethylation by histone methyltransferases (HMT) and histone demethylases (HDM) at lysine or arginine residues show different effects on gene activity depending on number and position of methyl groups. **d** Histone ubiquitinylation (Ub) at lysine residues alters histone structure and allows access of enzymes involved in transcription. **e** Histone phosphorylation (P) at distinct serine residues is known to be associated with increased gene expression, and it is also involved in DNA damage response and chromatin remodelling. Phosphorylation at linker histone (LH) H1 is considered to be a signal for the release of histone H1 from chromatin. In general, epigenetic regulation depends on the addition of epigenetic marks by writer enzymes (e.g. DNMT, HMT, HAT) and the removal of these marks by epigenetic eraser enzymes (e.g. HDAC and HDM) as well as epigenetic reader enzymes (not shown in this figure)

Even though the cells of an organism share the same set of genes, they are differentiated into diverse types of cells and tissues individually characterized by their own biochemical capabilities, (functional) morphology, and gene expression profile. Thus, there is a need for highly ordered regulatory mechanisms determining the fate of each cell. Epigenetic changes are heritable modifications affecting gene expression without causing alterations in the nucleotide sequence itself [13]. The most common epigenetic modifications (Fig. 1) are changes in the DNA methylation pattern, posttranslational histone modifications, and variations in the expression of non-coding microRNA (miRNA). DNA methylation is catalysed by DNA methyltransferases (DNMT), and histone acetylation state is adjusted by opposing activities of histone acetyltransferases (HAT) and histone deacetylases (HDAC). Histone methylation is regulated by histone methyltransferases (HMT) and histone demethylases (HDM) [4]. Of note, aberrant expression or activity of HMT [14, 15] and HDM [16] has also been associated with cancer development [4]. MiRNA is another epigenetic regulatory system that post-transcriptionally influences the regulation of gene expression and is important for RNA-silencing [17, 18]. Epigenetic changes have been reported during cancer development and are found in genes involved in cell differentiation, proliferation, and survival or apoptosis [4, 19]. CpG dinucleotides (cytosine-phosphate-guanine; cytosine nucleotide followed by a guanine nucleotide) are prone to methylation in the human genome. About 70 % of the CpG are methylated (mostly CpG dinucleotides, which are dispersed throughout the genome), whereas a minority of CpG residues is unmethylated (mostly CpG clusters also known as CpG islands, which are mainly located at the 5′ side of genes) [5, 12]. Methylation of CpG islands plays an important role in regulation of gene expression [4, 19]. Approximately 70 % of CpG islands in the human genome are resistant to de novo methylation caused by overexpression of DNMT1 [20], and even though 50 % of all mammalian genes exhibit CpG islands, only a minority is prone to silencing by hypermethylation [5, 20, 21]. Human DNMT (DNMT1, DNMT2, DNMT3a and DNMT3b) specifically methylate the C5 position of cytosine in CpG dinucleotides [5, 22]. DNMT1 maintains DNA methylation patterns, whereas DNMT2 shows only weak methylation activity [23]. DNMT3a and DNMT3b are responsible for de novo methylation of DNA essential during embryogenesis and other cell differentiation events [20, 23]. Most cancer cells exhibit on the one hand a global DNA hypomethylation and on the other hand, simultaneously, a DNA hypermethylation of specific promoter regions for e.g. tumour suppressor genes or genes important for apoptosis [5, 6]. Malignantly transformed cells modify transcription of genes by changes in the methylation of CpG islands within gene promoter regions and/or by changing posttranslational histone modifications like histone deacetylation as well as distinct histone methylation patterns resulting in decreased gene transcription (Fig. 1). In cancer cells, DNMT1 is both able to maintain DNA methylation and to de novo-methylate DNA of tumour suppressor genes [24]. However, aberrant DNA methylation is not limited to cancer cells; abnormal DNMT expression is also linked to various diseases including depression, anxiety disorder, dementia, autism, cardiovascular diseases, obesity and type 2 diabetes [25–30].

Histone proteins are present in eukaryotic nuclei, where they facilitate the dense packing of DNA and thus play an essential role in the dynamic accessibility of DNA for transcription factors. In humans, there exist two major histone families: the linker histone (LH) and the core histones. Each histone subfamily comprises one or more different variants. The core histones (two each of H2A, H2B, H3, and H4) form an octameric structure called nucleosome, which is a basic element of DNA packaging and consists of 146 base pair units of DNA that are coiled around the octamer of such core histone proteins [12]. The LH H1 binds to the nucleosome and the linker DNA helping to stabilize the chromatin fibre [31, 32]. Histone phosphorylation is mostly associated with actively transcribed genes, but phosphorylation of H1 is also considered to be an important signal for the release of the LH. The dynamic structure of chromatin allows rapid changes in gene regulation [5]. Moreover, the N-termini of histone proteins contain multiple lysine residues and are accessible to covalent modifications such as acetylation, methylation, sumoylation, biotinylation, phosphorylation, glycosylation, and ADP-ribosylation, thus allowing regulation of gene transcription (Fig. 1) [4, 5, 33–36]. Chromatin consists of nucleosome units connected by linker DNA, condensing the volume of the genetic information in eukaryotes [4, 37, 38]. Generally, two distinct states of chromatin are distinguished [4, 38]: heterochromatin is densely compacted and transcriptionally almost inactive; the decondensed euchromatin is only lightly packed, allowing transcriptional activity. Gene expression is hence determined by interactions between DNA methylation, histone modification, and nucleosome positioning influencing chromatin structure. Presence of chromatin remodellers, chromatin-associated proteins, and methyl DNA binding proteins are also important for structural modification of chromatin [4, 39, 40].

Histone acetylation is one of the most studied posttranslational histone modifications to date. Historically, HAT were divided into two groups: type A exhibits a nuclear localization, whereas type B is distributed throughout the cytoplasm and acetylates newly synthesized proteins [5, 33]. To date, three main families of HAT proteins are distinguished among others (MYST, GNAT, and CBP/p300) [4]. Based on their sequence homology to the enzymes found in yeast, HDAC are divided into four classes [41–43]: class I, which comprises the HDAC isoforms HDAC1, HDAC2, HDAC3, and HDAC8, is located predominantly in the nucleus; class IIA, containing HDAC4, HDAC5, HDAC7, and HDAC9 as well as class IIB including HDAC6 and HDAC10 (HDAC6 with two catalytic sites), preferentially shuttle between the nucleus and the cytosol; class III are the nicotinamide adenine dinucleotide (NAD^+^)-dependent sirtuins (SIRT1-7); and class IV consists only of HDAC11, which has catalytic residues in its active centre shared by class I and II [5, 41, 44, 45]. Class I, II, and IV HDAC exhibit homology in both sequence and structure and require a zinc ion for their catalytic activity. These three classes of HDAC completely differ from the sirtuin family regarding sequence and structure. Sirtuins were investigated for their contribution to lifespan prolongation under caloric restriction conditions in lower organisms [4, 46]. In addition, sirtuins exert a variety of different effects on DNA repair mechanisms, chromosomal integrity, cellular senescence, cell cycle progression, and transcriptional activity of tumour-associated proteins such as p53, p73, retinoblastoma protein (pRb), nuclear factor kappa-light-chain-enhancer of activated B cells (NF-κB), and the FoxO family [4, 46–48]. Different HDAC isoforms are frequently overexpressed in certain tumour entities, whereas reduced levels of specific HDAC isoforms were observed in some tumour types [4]. Therefore, it is still a big issue that many HDAC inhibitors (HDACi) show only little specificity among the different HDAC isoforms [49] and histone hyperacetylation does not necessarily correlate with regions of increased gene expression [36]. Nonetheless, HDACi seem to be promising cancer-preventive and therapeutic agents capable to reactivate tumour suppressor genes [50, 51]. Genomic instability found in majority of cancer cells causes an increased vulnerability against DNA damaging agents [50]. Therefore, tumour cells might be more susceptible to exogenous compounds causing oxidative stress by production of reactive oxygen species than healthy tissue [52]. Noteworthy, DNA and histone modifying enzymes are highly dependent on essential metabolites such as acetyl-CoA, iron, ketoglutarate, NAD^+^, and S-adenosyl methionine (SAM) and rely on a stable cellular metabolic state [12].

### Targeting cancer with epigenetically active compounds

#### DNA methyltransferase inhibitors in clinical use for the treatment of cancer

A number of DNMT inhibitors (DNMTi) are already in clinical use or currently under investigation in clinical trials. The pyrimidine nucleoside analogues azacitidine (5-azacytidine, Vidaza^®^) and decitabine (5-aza-2′deoxycytidine, Dacogen^®^) are approved by the US Food and Drug Administration (FDA) for the treatment of myelodysplastic syndrome (MDS) and acute myeloid leukaemia (AML) [4, 53, 54]. How DNMTi specifically affect cancer cells is currently under intensive investigation. The ribonucleoside azacitidine is an analogue of cytosine, which is important for DNA and RNA synthesis [55]. Azacitidine incorporates into RNA and to a lesser extent into DNA [56, 57]. Inclusion into RNA can subsequently cause disassembly of polyribosomes [58, 59], thus disturbing tRNA and ultimately protein synthesis, while incorporation into DNA induces covalent binding to DNMT, thus preventing DNA methylation and very likely replication. Noteworthy, the unspecific disruption of intracellular protein biosynthesis is probably responsible for the high toxicity of azacitidine. In contrast, decitabine is exclusively incorporated into DNA [60], acting as a DNMTi due to covalent binding [57, 61]. Decitabine is supposed to be a more potent DNMTi in vitro than other nucleoside and non-nucleoside inhibitors [62, 63]. Clinical efficacy in the treatment of AML, acute lymphocytic leukaemia (ALL), chronic myeloid leukaemia (CML), and chronic myelomonocytic leukaemia (CMML) is suggested to be caused by DNA demethylating activity of decitabine [64]. Generally, the identification of demethylation events caused by the use of DNMTi in vivo is challenging and shows substantial differences in patients [57, 65]. The majority of studies on humans have investigated the methylation state of the p15 tumour suppressor gene, which is often hypermethylated in MDS and AML. Unfortunately, demethylation and re-expression of p15 could not be correlated to the clinical responses of treated patients [57]. Limitations for the clinical application of the nucleoside inhibitors are their cytotoxicity (especially azacitidine), low drug stability, poor oral availability, and rapid elimination [5, 10]. Although several nucleoside inhibitors have demonstrated anticancer properties in preclinical models and are under clinical investigation, so far, partly due to their toxicity, only azacitidine and decitabine have been approved by the FDA for the treatment of cancer. Distinct flavonoids and other natural compounds like vitamin C (at high doses such as 8 mmol/L, achievable in patients by i.v. administration) [66] are known to act as DNMTi either directly by interaction with the active site of these enzymes or by indirect mechanisms. Therefore, flavonoids and their derivatives may serve as novel and alternative compounds with DNMTi activity for the prevention and treatment of cancer.

#### Histone deacetylase inhibitors in clinical use for the treatment of cancer

HDACi can be divided into different chemical classes. HDACi share a metal binding domain enabling them to compete with substrates for the required Zn^2+^-interaction in the binding pocket of the enzymes [5, 33]. Known HDACi are short chain fatty acids (e.g. sodium butyrate and valproate) with in vitro IC_50_ in the millimolar range, the very potent hydroxamic acids (e.g. trichostatin A (TSA) and vorinostat) displaying IC_50_ in nano- to micromolar range, benzamides (e.g. MS-275 and *N*-acetyldinaline), and cyclic tetrapeptides/epoxides (e.g. trapoxin and romidepsin) with IC_50_ in the nanomolar range [44, 67]. Noteworthy, some HDACi were isolated from natural sources or developed from plant-derived compounds. TSA is an antibiotic isolated from *Streptomyces sp.* with fungicidal properties and one of the most potent HDACi known to date with an IC_50_ in the low nanomolar range. Its production is expensive and TSA displays several undesirable side effects [5, 67]. Vorinostat and romidepsin progressed to clinical trials and are the first HDACi approved by the FDA for the treatment of cutaneous T cell lymphoma. Vorinostat is a chemical compound suitable for docking into the active site of HDAC of classes I, II and IV [5, 68]. Romidepsin is a natural product obtained from *Chromobacterium violaceum* and can be considered as a prodrug [51]. More recently, the HDACi belinostat and panobinostat were FDA approved. Belinostat is used in the treatment of relapsed or refractory peripheral T cell lymphoma, and panobinostat is available for the treatment of recurrent multiple myeloma in combination with bortezomib and dexamethasone [69, 70]. Despite the promising potential of HDACi in the treatment of some cancer entities, there have also been issues concerning low specificity to the different HDAC isoforms and adverse effects [36]. In this regard, some promising inhibitors like TSA were not approved for the clinical use and an intensive search for novel epigenetic drugs combined with the evaluation in clinical trial is ongoing [71]. Interestingly, some innocuous phytochemicals including flavonoids display a HDACi activity.

### Flavonoids

**Fig. 2:**
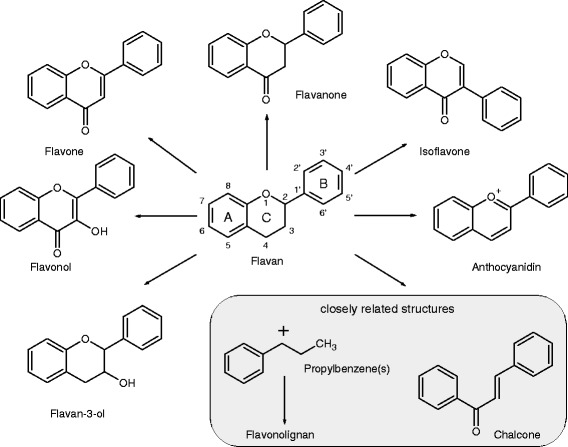
Chemical structures of flavonoid subclasses. The six flavonid subclasses include flavan-3-ols, flavonols, flavones, flavanones, isoflavones, and anthocyanidins, which all feature a flavan backbone. The closely related chalcones (ring closure causes flavonoid formation) and flavonolignans (complex flavonoids consisting of a flavonoid covalently bound to a lignan) are displayed as well (*grey box*)

Flavonoids are a large group of secondary plant metabolites (also known as phytochemicals). More than 4000 different flavonoids are described so far [72]. Flavonoids belong to the polyphenol family. The basic chemical structure of flavonoids is the flavan backbone. It comprises two phenolic rings (named A and B) linked by an oxygen-containing heterocycle (C) and is the structural feature shared by all flavonoids (Fig. 2). Depending on their chemical structure, flavonoids are divided into six subclasses: flavan-3-ols (also known as flavanols or catechins), flavonols, flavones, flavanones, isoflavones, and anthocyanidins [48]. Variations in the saturation, hydroxylation and glycosylation of the rings are responsible for the large number of individual compounds within each of these subclasses. Flavonoids are widely distributed throughout the plant kingdom, where they function as pigments, phytohormones, and protect against UV radiation, insect pests, and plant diseases [73, 74]. Furthermore, flavonoids have been reported to exert a number of biological activities in mammals, such as antibacterial, antiviral, analgesic, antiallergic, hepatoprotective, cytostatic, apoptotic, oestrogenic and anti-oestrogenic functions, to name only a few [75–77]. These diverse biological activities have been attributed to many molecular mechanisms, including the modulation of the activities of phase I and II detoxification enzymes, direct and indirect antioxidant activities [76, 78, 79], inhibition of protein kinases, effects on cell cycle, modulation of gene transcription, and epigenetic activities [5, 80]. The described effects are important due to the presence of these compounds in the human diet and their regular ingestion. According to a French study, published by Brat and colleagues, the total polyphenol intake from fruit is about three times higher compared to that from vegetables, based on the overall low polyphenol level in vegetables [81]. The mean total daily polyphenol intake of French adults is estimated to be 1193 ± 510 mg/day [82]. The flavonoid intake of the Australian population e.g. is estimated to be 454 mg/day with 92 % flavan-3-ols [83]. The estimated mean daily total flavonoid intake in US adults e.g. is described by Chun and coworkers with 189.7 mg/day, with a portion of 83.5 % from flavan-3-ols, 7.6 % flavanones, 6.8 % flavonols, 1.6 % anthocyanidins, 0.8 % flavones, and 0.6 % isoflavones [84].

### Epigenetic activities of flavonoids

#### Flavan-3-ols

**Fig. 3:**
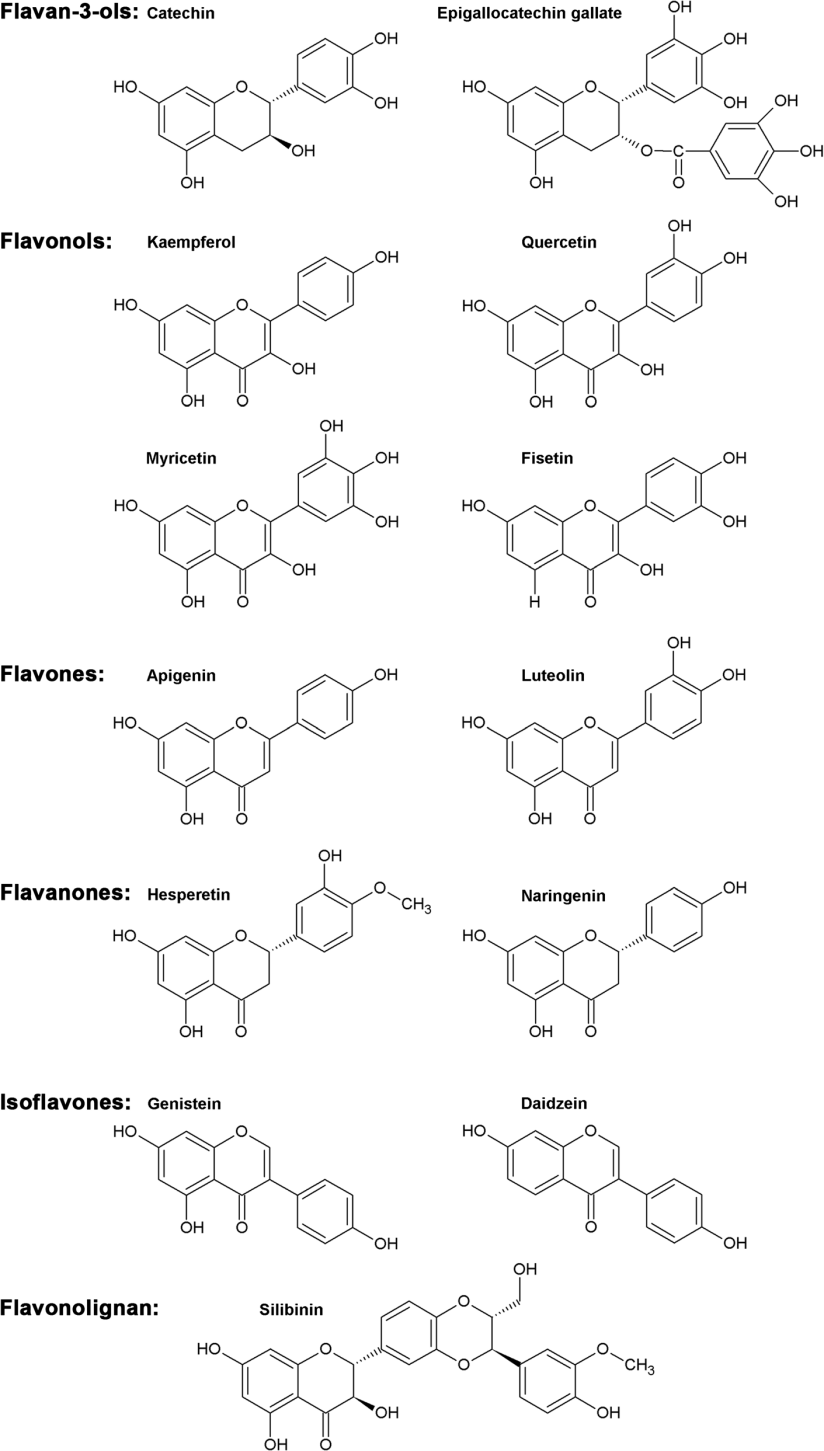
Chemical structures of flavonoids known to exert epigenetic activity. Flavan-3-ols: (+)-catechin and epigallocatechin-3-gallate; flavonols: kaempferol, quercetin, myricetin, and fisetin; flavones: apigenin and luteolin; flavanones: hesperetin and naringenin; isoflavones: genistein and daidzein; flavonolignan: silibinin

Rich sources of flavan-3-ols are e.g. apples, grapes, wine, cacao, and tea [72] (Fig. 2). Tea is the most widely consumed beverage worldwide next to water [85, 86]. Green, black, and oolong tea, which differ by their degree of fermentation, are the most popular teas. Green tea leaves are only dried and roasted, oolong tea is partially fermented, and black tea extensively fermented [87–90]. All of these teas contain varying amounts of e.g. (+)-catechin, (−)-epicatechin (EC), (−)-epicatechin-3-gallate (ECG), (−)-epigallocatechin (EGC), and (−)-epigallocatechin-3-gallate (EGCG). The flavan-3-ol EGCG is the most abundant flavonoid in green tea [5, 90, 91] (Fig. 3). EGCG induces apoptosis (e.g. in HaCaT, L5178Y, and DU 145 carcinoma cells, but not normal human keratinocytes) [92] and cell cycle arrest in A-431 human epidermoid carcinoma cells [92] while reducing oxidative stress (e.g. metal ion chelation by vicinal dihydroxyl and trihydroxyl structures) and angiogenesis [87, 93]. EGCG was further shown to inhibit DNMT activity by direct enzyme interaction leading to decreased cellular concentrations of 5-methylcytosine; contrariwise, Stresemann and coworkers reported no effect of EGCG on DNMT activity (2–50 μmol/L) in cancer cells after a 3-day treatment [5, 94]. It was described that Mg^2+^ enhances the inhibitory effect of EGCG on human DNMT activity [5, 95]. In the context of human cell lines, EGCG (20 μmol/L) inhibits DNMT activity in oesophageal (KYSE-150), colon (HT-29), prostate (PC-3), and breast (MCF7 and MDA-MB-231) cancer cells [2], and also other tumour entities [96–98]. An indirect inhibition of DNMT by flavan-3-ols is mediated by an increase in S-adenosyl-l-homocysteine (SAH). This increase in SAH may be caused by catechol-O-methyltransferase (COMT) mediated methylation of these flavonoids. Tea catechin-mediated COMT inhibition was investigated in the context of rat, mouse, and human liver cytosol [99, 100]. SAH itself, which is formed out of the methyl donor SAM, is a potent inhibitor of DNMT [5, 101]. EGCG was found to contribute to the degradation of DNMT3a and HDAC3 in human HCT 116 colon cancer cells [102]. Interestingly, EGCG treatment of MCF7 and MDA-MB-231 breast cancer cells resulted in downregulation of human telomerase reverse transcriptase (hTERT) expression and thus decreased telomerase levels. As the telomerase is responsible for lengthening of telomeres in DNA strands, this enzyme is almost absent in normal cells, whereas it is considerably expressed in spontaneously immortalized cells such as cancer cells. Even though tea catechins are less potent DNMTi than the chemical nucleoside inhibitors, counted as non-nucleoside inhibitors, they could exert a stable and moderate DNMT inhibition due to long-term consumption of green tea. Catechins are also hypothesized to be more gene and tissue specific than the nucleoside inhibitors, although experimental evidence is lacking [5, 63]. Sustained consumption of tea may be a promising cancer-preventive or even cancer-therapeutic approach [5]. In addition, EGCG modulates cellular HAT activity and thus histone acetylation and thereby alters the chromatin structure [50, 103]. EGCG (100 μmol/L) was found to inhibit the majority of HAT enzymes confirmed by colourimetric HAT activity assay and in vitro fluorography analyses of HAT enzymes [5, 103]. Moreover, EGCG also modulates miRNA expression in hepatocellular carcinoma cells [87], which further contributes to its broad spectrum of epigenetic activities. Noteworthy, flavan-3-ols exert further molecular effects, which could contribute to their inherent anticancer activity, such as inhibition of dihydrofolate reductase leading to reduced cellular levels of folic acid [104]. Several in vivo studies investigated the epigenetic modulation mediated by flavan-3-ols, especially ECGC, and their impact on cancer formation and tumour growth. Henning and coworkers showed that substitution of drinking water with brewed green tea (about 700 mg/L total green tea catechins) using male severe combined immunodeficiency (SCID) mice with human LAPC4 prostate cancer xenografts [105] resulted in a reduced tumour volume. The results of the prostate cancer model indicate that green tea inhibited mRNA and protein expression of DNMT1 in the tumour cells, which in turn reactivated antioxidative enzymes [105].

Another interesting study that focuses on colorectal carcinogenesis in the azoxymethane-Apc^Min/+^ mouse model investigated green tea as well. In comparison to the water control group, administration of green tea solution (0.6 % *w*/*v*), as only source of beverage, reduced the number of newly formed tumours significantly. Moreover, this study detected a green tea-mediated rise in protein and transcript levels of retinoid X receptor alpha (RXRa), which was found to be selectively downregulated in intestinal tumours of control mice. Analyses of CpG sites at the promoter region of the RXRa gene illustrated that consumption of green tea decreased CpG methylation [106]. Therefore, the authors suggested that in this in vivo intestinal cancer model, the demethylating activity of the green tea solution decelerates or even prevents tumourigenesis.

Instead of oral administration, the effects of flavonoids on tumour formation by topical application were also investigated by in vivo studies. EGCG in a hydrophilic cream as vehicle was tested for its antiphotocarcinogenic activity using a SKH-1 hairless mouse model [107]. Measuring tumour incidence and tumour size, the topical application of ECGC displayed a pronounced protection against photocarcinogenesis [107]. Moreover, the transformation of papillomas to carcinomas by ultraviolet B (UVB) seemed to be inhibited by EGCG as well [107].

Despite these promising and interesting findings for green tea, some studies demonstrated that the demethylating activity is not always detectable in an in vivo situation. For instance, the oral consumption of green tea polyphenols (0.3 % in water) by transgenic adenocarcinoma of the mouse prostate (TRAMP) mice with an age of 4 weeks did not affect the DNA methylation of prostate, gut or liver. Moreover, the administration of green tea polyphenols did not inhibit tumour progression in this murine prostate model [108, 109].

#### Flavonols

Flavonols are abundant in vegetables and fruits, among which onions represent a rich dietary source (Fig. 2). Other important dietary sources for flavonols include tea, apples, berries, and wine. Quercetin is the predominant flavonol [110] (Fig. 3). Bioavailability of quercetin was extensively investigated compared to most of the other flavonoids [111]. It seems that the glycosylation state of quercetin strongly influences its intestinal absorption rate [72]. While both, the glycosides and aglycone of quercetin are absorbed, the bioavailability of the glycosides is much better [72]. Due to its long elimination half-life in vivo [72], daily intake (e.g. 1 week ingestion of a diet rich in onion) resulted in an accumulation and an increase of quercetin from 0.04 ± 0.04 μmol/L before to 0.63 ± 0.72 μmol/L [112–114]. Fisetin is found in strawberries, apples, onions, wine, and tea [46, 115], whereas grapes, berries, red wine, and tea are sources of myricetin [116, 117] (Fig. 3). Kaempferol is found in fruits and vegetables such as tomatoes, hop, red grapes, grapefruit, strawberries, and *Gingko biloba* [118] (Fig. 3). The flavonols quercetin, fisetin, and myricetin were tested for DNMT inhibition and their IC_50_ values were determined: quercetin (1.6 μmol/L), fisetin (3.5 μmol/L), and myricetin (1.2 μmol/L) [2, 95]. All three flavonols inhibited DNMT1-mediated DNA methylation in a concentration-dependent manner [95]. Myricetin, the flavonol exerting the strongest DNMT inhibition, has a pyrogallol moiety (structurally related to benzene with three vicinal hydroxy groups) similar to the gallic acid moiety of EGCG [5, 95]. It has also been shown that the two flavonols quercetin and fisetin activate sirtuins [5, 80], whereas quercetin (100 μmol/L) seems to drive both HAT activation and HDAC inhibition determined with a colorimetric activity assay, which does not distinguish between HAT or HDAC isoforms [119]. Consistent with these findings, increased histone H3 acetylation was described after quercetin exposure of leukaemia HL60 cells [50]. Noteworthy, investigation of the chemopreventive and therapeutic capabilities of quercetin in a 7,12-dimethylbenz[a]anthracene (DMBA)-induced hamster buccal pouch (HBP) carcinoma model showed positive correlation between inhibition of HDAC1 and DNMT1 and anticancer effects exerted by quercetin, such as induction of cell cycle arrest and apoptosis as well as inhibition of angiogenesis and invasion. If quercetin was administered at the same time with DMBA, both the tumour incidence and the tumour burden were decreased. Treatment with quercetin after DMBA exposure significantly slowed tumour growth [120]. The flavonol kaempferol exhibits also a distinct inhibitory activity towards HDAC enzymes. In particular, our group observed a hyperacetylation of histone H3 by kaempferol (visualized by Western blot of hyperacetylated H3 after a 24-h treatment of human hepatoma cell lines HepG2 and Hep3B as well as the colon cancer cell line HCT 116). This effect seemed to depend on inhibition of class I, II, and IV HDAC enzymes (HDAC1-11) [118]. In addition, reduced cellular viability and proliferation was demonstrated for HepG2, Hep3B, and HCT 116 cancer cells after incubation with kaempferol concentrations that mediated HDACi activity [118].

#### Flavones

Celery, chamomile, and parsley are rich sources of apigenin [103] (Figs. 2 and 3). Carrots, peppers, celery, olive oil, peppermint, thyme, rosemary, and oregano are important dietary sources of luteolin [121] (Fig. 3). Apigenin and luteolin exert inhibitory effects on 5-cytosine DNMT as shown, using nuclear extracts of KYSE-510 cells [122]. Both flavones were tested at concentrations of 20 and 50 μmol/L. Luteolin showed a more pronounced inhibition of DNMT enzymes with an efficacy of about 50 % at 50 μmol/L, while apigenin only displayed a 35 % inhibition at 50 μmol/L [122]. Apigenin causes cell cycle arrest and apoptosis in human prostate cancer cells (PC-3 and 22Rv1) [123]. The latter study revealed that apigenin-mediated growth inhibition is due to inhibition of class I HDAC. The authors showed that treatment of PC-3 and 22Rv1 cells with 20–40 μmol/L apigenin inhibited HDAC1 and HDAC3 on mRNA and protein levels resulting in a global histone H3 and H4 hyperacetylation. Further experiments in a PC-3 xenograft model of athymic nude mice showed that oral intake of apigenin at a constant daily dose of 20 or 50 mg per mouse for 8 weeks inhibited HDAC activity and expression of HDAC1 and HDAC3 in the cancer tissue and resulted in reduced tumour growth [123].

#### Flavanones

Two flavanones tested for epigenetic activity are hesperetin and naringenin (Figs. 2 and 3). Flavanones are abundant in the peel of citrus fruits, which are not directly ingested in significant amounts, and thus the major dietary sources for flavanones are citrus juices [124, 125]. Both hespertin and naringenin inhibit DNMT activity in nuclear extracts of human oesophageal squamous cell carcinoma KYSE-510 cells incubated for 1.5 h with 20 or 50 μmol/L. DNMTi activity was not specified for particular isoforms, and hesperetin was found to be the more potent inhibitor of both tested flavanones [122].

#### Isoflavones

Isoflavones are found e.g. in soybeans, fava beans, and kudzu [87] (Fig. 2). Genistein and daidzein, the most prominent and well-characterized isoflavones are structurally similar to 17β-oestradiol, which allows their binding to oestrogen receptors and explains their, albeit weak, phyto-oestrogenic activity [126–128] (Fig. 3). Regarding the epigenetic activity of isoflavones, it was demonstrated that genistein, daidzein, and biochanin A inhibit DNMT enzymes [5, 122, 129]. Genistein (at concentrations of 2–20 μmol/L) reduced genomic DNA hypermethylation and subsequently increased the protein level of retinoic acid receptor β (RARβ), p16^INK4a^, and O^6^-methylguanine DNA methyltransferase (MGMT) in human KYSE-510 cells [129]. Further studies confirmed that genistein inhibits DNMT1, DNMT3a and DNMT3b, and leads to activation of silenced tumour suppressor genes [130]. A similar dose-dependent competitive and non-competitive inhibition of DNMT by reduction of the cellular availability of SAM was found with 20–50 μmol/L genistein in oesophageal squamous cell carcinoma KYSE-150 cells and the prostate cancer cells lines LNCaP and PC-3 [129]. Interestingly, genistein represses hTERT expression in breast cancer cells, paradoxically by promoter demethylation, which is similar to the activity of ECGC described above [131–133]. The epigenetic activity of genistein was also assessed in vivo. Maternal genistein diet of agouti mice during pregnancy was found to have long-term effects on offspring and altered coat colour due to epigenetic modulation. In this context it is interesting that prenatal exposure to genistein (270 mg/kg feed) not only leads to lifelong changes in the DNA methylation patterns but also has an impact on gene expression including in the haematopoietic lineage [134]. The epigenetic activity of dietary genistein in timed pregnant Sprague-Dawley rats and their male pubs was also investigated. Genistein was fed either as a soy protein lysate or purified compound (equivalent to 140 mg/kg genistein aglycone) and compared to a genistein-free control diet. Male pubs were injected with the chemical carcinogen azoxymethane, which decreases levels of acetylated histone H3. Colon samples were investigated 6 weeks after carcinogen injection. In this experimental setting, dietary genistein modulated the responses of wingless-related integration site (Wnt) genes by DNA methylation and histone modifications during carcinogen exposure. Thus, it was suggested that dietary genistein may prevent neoplastic development [135]. This emphasizes that genistein has multiple effects not only on DNMT enzymes but also on posttranslational histone modifications like histone demethylation, HAT activation, and SIRT inhibition [50, 135, 136]. Treatment of MCF7 and MDA-MB-231 breast cancer cells with genistein (18.5 μmol/L) and daidzein (78.5 μmol/L) decreased histone trimethylation and increased histone acetylation of six different genes each responsible for a protein associated with breast cancer (histone-lysine *N*-methyltransferase (EZH2), breast cancer 1, early onset (BRCA1), oestrogen receptor α (ERα), oestrogen receptor β (ERβ), nuclear receptor coactivator 3 (SRC-3), and P300) [137]. Genistein inhibited HDAC activity by 15 % at concentrations of 5–20 μmol/L in KYSE-510 cells. Although genistein is a less potent inhibitor of DNMT than EGCG, it is more stable in solution and the additional weak HDAC inhibitory activity of genistein was suggested to result in comparable gene reactivation as observed for EGCG [5, 122]. Thus, genistein-mediated gene reactivation may be based on a synergistic effect of DNMT and HDAC inhibition [5, 138]. Moreover, there is a strong body of evidence that genistein and other isoflavones regulate miRNA expression in cancer cells and hence have an impact on tumour growth [87, 133, 139]. Despite the promising preventive and therapeutic potential of genistein, there is also a debate on possible deleterious effects by genistein administration in the treatment of breast cancer and probably other hormone dependent cancer entities in humans [140]. Another report also described disadvantageous effects of isoflavones in regard to increased formation of metastases [141].

#### Anthocyanidins

Anthocyanidins are responsible for the blue and red colours of plants and are found e.g. in many berries, cherries, and grapes [72] (Fig. 2). Anthocyanidins are flavonoid aglycones; their glycosides are called anthocyanins. Well-known anthocyanidins include cyanidin, delphinidin, malvidin, and pelargonidin, to name only a few. To the best of our knowledge, the epigenetic activities of the anthocyanidins have not yet been investigated.

#### Flavonolignans

From a chemical point of view, flavonolignans are composed of a flavonoid fused to a lignan (Fig. 2). Silibinin is the most active flavonolignan and is found e.g. in a standardized extract from milk thistle known as sylimarin, which is an antidote used to prevent toxic liver damage upon ingestion of toxins (e.g. myrocristin-induced toxicity in mice, cases of mushroom poisoning in humans) [142–144] (Fig. 3). Silibinin contains two diastereomers named silybin A and B [145]. In a model of colon cancer progression with primary human colon adenocarcinoma cells SW480 and the corresponding metastatic derivative SW620, silibinin inhibited DNMT activity in both cell lines [146]. However, no effect on HDAC activity was observed, which contrasted with previous reports showing inhibitory effects of silibinin on HDAC enzymes [147–149]. Lah and coworkers used high concentrations of silibinin (120 μmol/L and 240 μmol/L, respectively) for 24 h to treat Huh7 human hepatoma cells and found significantly increased levels of acetylated histones H3 and H4 [147]. The effect of silibinin on human hepatocellular carcinoma xenografts in nude mice was assessed by Cui and colleagues. After subcutaneous inoculation of Huh7 cells, mice were treated with vehicle, 80 mg/kg or 160 mg/kg silibinin per day, respectively. A 5-week treatment with silibinin resulted in elevated levels of acetylated histones H3 and H4 in the tumour tissue and in reduced tumour growth [148]. Similar effects were reported for the non-small cell lung cancer cells H1299 and a corresponding xenograft mouse model (athymic (nu/nu) male nude mice were subcutaneously injected with H1299 cells and treated with 100 mg/kg silibinin per day for 4 weeks) [149]. The authors found inhibition of HDAC1, HDAC2, and HDAC3 resulting in increased levels of acetylated histones H3 and H4. Promising synergistic effects of silibinin with the well-known HDACi compounds TSA and vorinostat were also reported [149].

## Conclusions

### Epigenetic activities of flavonoids and their impact on human health

**Fig. 4:**
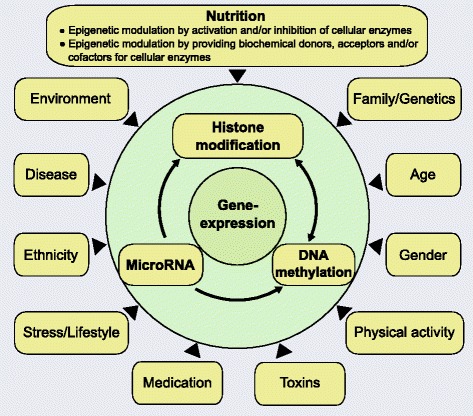
Modulation and interaction of epigenetic mechanisms. Gene regulation depends on a complex interplay between posttranslational histone modifications and DNA methylation. MiRNA either directly affect gene expression or modulate other epigenetic mechanisms. Epigenetic activity in general is influenced by several exogenous and endogenous factors including nutrition

A multitude of biological effects has been reported for flavonoids, including their influence on phase I and II detoxifying enzyme activity, antioxidant properties, protein kinase inhibition, cell cycle regulation, transcriptional, and epigenetic activities. Flavonoids have attracted increasing attention due to their diverse health-promoting effects. Especially the possible chemopreventive and antitumour activities of flavonoids and other natural compounds are noteworthy due the fact that cancer incidence and mortality is globally still exceptionally high [150]. Interestingly, the cancer incidence varies between the different regions of the world, and for decades observational studies have been suggesting that nutritive and lifestyle risk factors strongly contribute to this discrepancy (Fig. 4) [151–153]. According to this, it was currently estimated that many cancer-related deaths could be prevented by adequate lifestyle modifications, particularly changes in diet or nutrition [12]. The consumption of diets rich in fruits and vegetables has been consistently associated with a significantly reduced risk of cancer development with an impact on both cancer initiation and progression [87, 154–158]. Importantly, emerging evidence strongly suggests that diet is a major modulator of the epigenetic state of cells and is able to reverse abnormal or altered gene expression patterns [87]. This is in line with growing evidence for sustained remodelling of the human epigenome during lifetime by nutrition, exercise, mental pressure or stress and environment (Fig. 4) [159]. The importance of such acquired epigenetic patterns is supported by observations in monozygotic twins, where one is affected by several diseases such as diabetes mellitus, while the other remains unaffected [160]. Young monozygotic twin siblings seem to be epigenetically relatively similar to each other, whereas epigenetic changes and a different gene expression profile were found in aged twin pairs [160]. These differences are indicated to depend on environmental factors such as lifestyle and time spent together [160]. Greatest changes in DNA methylation patterns were observed in aged monozygotic twins if they did not share environments during lifetime [12]. According to epidemiological studies and dietary interventions in animal models, it was strongly suggested that nutrition-derived epigenetic modifiers influence the epigenome and even the cancer risk programming of unborn children in utero [2, 12]. There is also increasing evidence that maternal malnutrition and deranged metabolism could have detrimental effects on offspring (“fetal programming”) and it is very likely that epigenomes are continuously shaped during lifetime exerting effects also on subsequent generations [12]. Therefore, dietary factors, including certain flavonoids, may have beneficial effects on human health by remodelling of the epigenome and other modes of action. Representatives of the flavonoid subclasses flavan-3-ols (catechin and EGCG), flavonols (kaempferol, quercetin, myricetin, and fisetin), flavones (apigenin and luteolin), flavanones (hesperetin and naringenin), isoflavones (genistein and daidzein), and the flavonoid-related class of flavonolignans (silibinin) have been reviewed above focusing on DNA methylation and histone acetylation [2, 5, 95, 122, 146]. As flavonoids are present in fruits, vegetables, and tea, a short overview of food containing high amounts of these epigenetically active flavonoids is depicted in Table 1. Nonetheless, it remains controversial if e.g. long-term exposure to “epigenetic diets” rich in flavonoids or flavonoid supplements could also induce unwanted effects due to their lack of specificity regarding distinct isoforms of enzymes responsible for epigenetic modulation like HAT and HDAC [12]. Low specificity was found to limit the clinical application of epigenetic drugs like HDACi, and despite their epigenetic activity, flavonoids target a multiplicity of other pharmacological pathways. On the one hand, these pleiotropic effects such as inhibition of angiogenesis, inflammatory processes, and induction of apoptosis [161, 162] can be beneficial for the treatment of several diseases and in particular cancer where most of mono-therapeutic efforts are non-satisfying [163, 164]. On the other hand, there is also a risk to induce unwanted side effects, and there is little knowledge about their bioavailability (e.g. aglycones and glycosides), metabolism, and in particular about their bioactivity and the fate of arising metabolites. Moreover, many clinicians are generally incredulous towards phytochemicals in the treatment of life-threatening diseases, and appropriate animal studies and clinical studies with flavonoids are still very limited. In terms of cancer treatment, oncologists are sceptical towards the antioxidant effects exerted by flavonoids, especially when combined with classical cytostatic drugs. It is still not clear if antioxidant properties are a problematic issue during chemotherapy. Generally, the use of flavonoids especially at super-physiological doses as conducted in several in vitro studies has to be carefully evaluated for the in vivo situation. Moreover, most flavonoids and other polyphenols with epigenetic activity in vitro have not yet been tested in animal models, and when tested, only a few epigenetic marks have been analysed in depth. In addition, the overall functional relevance of epigenetic modulation exerted by flavonoids and other natural compounds in chemoprevention and chemotherapy has to be further elucidated [165].

**Table 1:** Food containing high amounts of epigenetically active flavonoids

Description	Class	Flavonoid	Ǿ mg/100 g	Sources of data
Grapefruit, raw (not specified as to colour) (*Citrus paradisi*)	Flavanones	Hesperetin	1.50	^a^USDA Database for the Flavonoid Content of Selected Foods: e.g. [193]
		Naringenin	53.00	
	Flavonols	Kaempferol	0.40	
		Quercetin	0.50	
Onions, red, raw	Flavones	Apigenin	0.24	^a^USDA Database for the Flavonoid Content of Selected Foods: e.g. [193–196]
		Luteolin	0.16	
	Flavonols	Kaempferol	0.70	
		Myricetin	2.16	
		Quercetin	39.21	
Soybeans, mature seeds, raw (all sources)	Isoflavones	Daidzein	62.07	^b^USDA Database for the Isoflavone Content of Selected Foods: e.g. [197–202]
		Genistein	80.99	
Spices, parsley, dried (*Petroselinum crispum*)	Flavones	Apigenin	4503.50	^a^USDA Database for the Flavonoid Content of Selected Foods: e.g. [196]
		Luteolin	19.75	
Strawberries (including frozen unsweetened strawberries)	Flavonols	Fisetin	16	[203]
		Kaempferol	0.49	^a^USDA Database for the Flavonoid Content of Selected Foods: e.g. [204, 205]
		Myricetin	0.35	
		Quercetin	0.46	
Cacao beans	Flavan-3-ols	(+)-Catechin	88.45	^a^USDA Database for the ^a^Flavonoid Content of Selected Foods: e.g. [206]
		(−)-Epicatechin	99.18	
Tea, black, brewed, prepared with tap water	Flavan-3-ols	(+)-Catechin	1.51	^a^USDA Database for the Flavonoid Content of Selected Foods: e.g. [196, 207–209]
		(−)-Epigallocatechin 3-gallate	9.36	
	Flavonols	Kaempferol	1.41	
		Myricetin	0.45	
		Quercetin	2.19	
Tea, green, brewed, decaffeinated	Flavan-3-ols	(−)-Epigallocatechin 3-gallate	26.05	^a^USDA Database for the Flavonoid Content of Selected Foods:
	Flavonols	Kaempferol	1.00	
		Myricetin	1.00	
		Quercetin	2.77	

Flavonoid and isoflavone content are summarized in the USDA databases cited below:

^a^Bhagwat, S., Haytowitz, D.B. Holden, J.M. (Ret.). 2013. USDA Database for the Flavonoid Content of Selected Foods, Release 3.1. U.S. Department of Agriculture, Agricultural Research Service. Nutrient Data Laboratory Home Page: http://www.ars.usda.gov/News/docs.htm?docid=6231

^b^Bhagwat, S., Haytowitz, D.B. Holden, J.M. 2008. USDA Database for the Isoflavone Content of Selected Foods, Release 2.0. U.S. Department of Agriculture, Agricultural Research Service, Nutrient Data Laboratory Home Page: http://www.ars.usda.gov/News/docs.htm?docid=6382

According to these uncertainties and hindrances, the discussion on epigenetic modulation by flavonoids and their impact on human health remains highly controversial and gives a possible explanation for the missing breakthrough of these natural compounds in clinical use [166–171].

### Intake of flavonoids and cancer risk

According to the currently available data, the intake of a balanced diet is strongly recommended for maintaining health and for the prevention of a broad spectrum of “Western civilization” diseases like cancer. Current studies reveal that malnutrition and obesity are under-recognized, but are important risk factors for cancer, cancer recurrence, and cancer-related mortality. Moreover, obesity negatively affects treatment outcome as well as prognosis of cancer patients [172]. Due to the significantly increased prevalence of obesity on a global scale, nutrition and lifestyle have become important aspects of cancer prevention and therapy. Therefore, many phytochemicals and/or their respective metabolites derived from fruits, vegetables or nutrients are currently analysed for their potential beneficial effects on health. In this respect especially, flavonoids as a large group of secondary plant metabolites have been under extensive investigation for decades [72]. Importantly, it has been controversially discussed if flavonoids exert any protection against cancer in humans. Epidemiological studies found conflicting results with respect to the effects of flavonoid intake and cancer incidence and mortality. For pancreatic cancer, a large prospective study with an analytic cohort of 537,104 persons found no evidence that flavonoids have a protective role in carcinogenesis (hazard ratio (HR) = 1.09) [173]. Another large prospective study with a cohort of 477,312 men and woman recruited in 10 European countries also found no association between total flavonoid intake and bladder cancer risk [174]. In contrast, some experimental data suggest that specific flavonoids could even promote tumour formation in certain subsets of patients. A randomized placebo-controlled study on female patients illustrated that the supplementation of soy, which contains high amounts of isoflavones, may upregulate genes that drive cell cycle and proliferation pathways and therefore could adversely affect breast carcinogenesis [175]. In this regard, a large prospective cohort study with a multiethnic population of 84,450 women examined the potential connection of dietary isoflavone intake and overall breast cancer risk. Interestingly, no statistically significant association between dietary isoflavone intake and overall breast cancer risk was found (HR = 0.96) [176]. In the same study, some ethnic groups benefited from a high isoflavone intake and had a reduced risk in breast cancer incidence, which is in line with a meta-analysis of corresponding prospective studies (relative risk (RR) = 0.89 on average with RR = 0.76 for Asian women and only RR = 0.97 for Western women) [177]. Although the impact of specific flavonoids on particular tumour entities is discussed controversially in the literature, the overall evidence suggests that flavonoids rather protect from cancer.

Referring to this, the cancer-preventive potential of dietary flavonoids was recently reviewed by Romagnolo and Selmin [178]. Epidemiological studies suggest that flavonoid ingestion reduces the risk of versatile cancer entities like pancreas, prostate, lung, colon, breast, and prostate cancer even though results are sometimes inconclusive [178]. It is challenging to construe data obtained from epidemiological studies because the majority are case-control or retrospective studies and less prospective trials, respectively [178].

In this regard, a prospective study involving 9959 men and women observed a reduction in lung cancer cases associated with increased flavonoid intake over a period of 24 years (RR = 0.54) [179].

Focusing on the subclasses of flavonoids, in an Italian case-control study with about 10,000 cancer patients and 16,000 controls, the risk to develop oral or laryngeal cancer was inversely related to the intake of total flavonoids (odds ratio (OR) = 0.56 and 0.60, respectively), flavanones (OR = 0.51 and 0.60, respectively), and flavonols (OR = 0.62 and 0.32, respectively) [178, 180]. Comparable results for laryngeal cancer were reported for flavanones (OR = 0.60), flavan-3-ols (OR = 0.64), and flavonols (OR = 0.32) [181], whereas no association was found for isoflavones, anthocyanidins, and flavones [178]. Flavanone intake mainly by consumption of citrus fruits reduced oesophageal cancer risk (OR = 0.38) [178, 182]. Protective effects against gastric cancer were reported for quercetin and EGCG [183, 184], whereas an increased risk for higher isoflavone intake was described in Japanese women simultaneously using exogenous hormones [185]. Total flavonol and particularly kaempferol intake was reported to reduce the risk of pancreatic cancer (OR = 0.78) [186].

In line, the large prospective study of Zamora-Ros and colleagues suggested a protective association between dietary intake of flavonols and the risk of bladder cancer (HR = 0.74) [174]. Two large studies analysing the risk of epithelial ovarian cancer concluded that several flavonoids and in particular flavonols seem to be associated with a reduced risk of ovarian cancer (HR = 0.85 for total flavonoids and RR = 0.75 for the combined intake of myricetin, kaempferol, quercetin, luteolin, and apigenin) [187, 188]. For isoflavones (another subclass of flavonoids), a randomized placebo-controlled trial displayed that uptake of isoflavones could reduce the prostate cancer risk [189]. These results are in line with the observation that supplementation of isoflavonoids seems to diminish the increase of serum prostate-specific antigen (PSA) in men following local therapy [190]. Even though there are obvious study limitations based on diet and lifestyle variables, several trials detected an association between dietary intake of specific flavonoids or flavonoid subclasses and reduced occurrence of certain cancer types. The molecular mechanisms underlying the anticancer properties of flavonoids are numerous and include the modulation of cellular signalling pathways, direct and indirect antioxidant activities, and epigenetic phenomena. The latter are important for tumour formation and growth and thus also for cancer prevention and therapy. Flavonoids are epigenetically active both in vitro and in vivo, albeit very high and often non-physiological concentrations have been used in experiments to investigate their epigenetic activities. Nevertheless, some flavonoids, such as quercetin, accumulate in blood and tissues over time [72, 112] and may thus reach sufficiently high concentrations to exert epigenetic activity. Furthermore, flavonoids may act synergistically with each other, with other natural compounds present in the diet and/or with synthetic drugs, and may thereby facilitate changes to the epigenome at low concentrations [191]. Nonetheless, low bioavailability of flavonoids is a crucial issue (due to restricted intestinal digestive and absorptive dynamics, metabolism by the intestinal microflora and after absorption) [5, 192]. Further in-depth research and prospective as well as mechanistic studies are required to investigate the beneficial effects of the different flavonoids in detail. Based on these data, simple changes in diet and food intake could contribute to prevention and treatment of human diseases including cancer. Safe, effective, and especially pleiotropic chemopreventive compounds are urgently needed considering the fact that cancers often exhibit long latencies of about 20 years and that detrimental changes in malignant cells should be counteracted as early as possible [12]. Many flavonoids and other polyphenols seem to regulate multiple targets e.g. involved in cancer-inflammation and could therefore be low priced, easily available, and highly tolerable compounds, and long-term exposure at physiological concentrations could shape the epigenome in a desirable and cumulative manner [12]. Despite the promising results for diets rich in fruit and vegetables in terms of disease prevention, it remains unclear if additional supplementation with isolated flavonoids would have significant additional beneficial health effects in humans. Clearly, pharmacological drugs are inevitably required for cancer therapy, but it seems that flavonoids and other natural compounds could contribute to future treatment strategies.

## Abbreviations

ALL: acute lymphocytic leukaemia
AML: acute myeloid leukaemia
Apc: adenomatosis polyposis coli
BRCA1: breast cancer 1, early onset
CML: chronic myeloid leukaemia
CMML: chronic myelomonocytic leukaemia
COMT: catechol-O-methyltransferase
DMBA: 7,12-dimethylbenz[a]anthracene
DNMT: DNA methyltransferase
DNMTi: DNA methyltransferase inhibitor
EC: (−)-epicatechin
ECG: (−)-epicatechin-3-gallate
EGC: (−)-epigallocatechin
EGCG: (−)-epigallocatechin-3-gallate
ERα: oestrogen receptor α
ERβ: oestrogen receptor β
EZH2: histone-lysine *N*-methyltransferase gene
FDA: US Food and Drug Administration
FoxO: forkhead-box-protein O
GNAT: GCN5 *N*-acetyltransferase
HAT: histone acetyltransferase
HBP: hamster buccal pouch
HDAC: histone deacetylase
HDACi: histone deacetylase inhibitor
HDM: histone demethylase
HMT: histone methyltransferase
HR: hazard ratio
hTERT: human telomerase reverse transcriptase
IC_50_: half maximal inhibitory concentration
LH: linker histone
MDS: myelodysplastic syndrome
MGMT: O^6^-methylguanine DNA methyltransferase
miRNA: non-coding microRNA
MYST: MOZ/YBF2/SAS2/TIP60
NF-κB: nuclear factor kappa-light-chain-enhancer of activated B cells
OR: odds ratio
p16^INK4a^: cyclin-dependent kinase inhibitor 2A, multiple tumour suppressor 1
p53: tumour protein p53
pRb: retinoblastoma protein
PSA: prostate-specific antigen
RARβ: retinoic acid receptor β
RR: relative risk
RXRa: retinoid X receptor alpha
SAH: S-adenosyl-l-homocysteine
SAM: S-adenosyl methionine
SIRT: sirtuin
SRC-3: steroid receptor coactivator-3
TRAMP: transgenic adenocarcinoma of the mouse prostate
TSA: trichostatin A
UVB: ultraviolet B
Wnt: wingless-related integration site
*w*/*v*: mass/volume (mass concentration)
